# The impact of digital economy development on coal consumption: Evidence from Chinese provinces

**DOI:** 10.1016/j.heliyon.2024.e33780

**Published:** 2024-06-27

**Authors:** Qijing Xiong, Peter Chan, Fan Bai

**Affiliations:** a School of Economics and Management, Sichuan Minzu College, Kangding, 626001,China; b School of Digital Economics,Sichuan University Jinjiang College, Meishan, 620860, China; c School of Economics and Management, Sichuan Tourism University, Chengdu, 610000, China

**Keywords:** Digital economy development, Coal consumption, Financial technology (fintech), Technological innovation quality

## Abstract

This study investigates the impact of the development of the digital economy (DE) on coal consumption and the underlying mechanisms using Chinese provincial data over the period 2011–2022. The results demonstrate that DE development is correlated with reduced coal consumption. When the DE development increases by one standard deviation, the reduction in coal consumption is 0.1938. The underlying mechanism is attributed to the DE facilitating the financial technology development and technological innovation's quality, which are conducive to reducing coal consumption.

## 1 Introduction

Coal, which is known as “black gold,” is a key facilitator of economic development [1]; however, following more than 30 years of rapid economic growth, China's coal resources are becoming increasingly depleted [2]. Since 2008, technologies such as internet, cloud computing, artificial intelligence (AI), blockchain, big data, and internet of things have been deeply integrated with China's economy, advancing digital economy (DE) development, which improves production efficiency and functions as a new driver of economic development. Therefore, the effects of DE development (as a new kinetic energy) on coal consumption are of interest.

Existing studies indicate that the economic impact of DE is based on information and digital knowledge, which are core production factors, and relies on modern information networks, which serves as the important carriers. The influence of DE is extensive. Bitcoin, a product of the DE, is becoming a distinct alternative investment and asset class worldwide [3]. Information and Communications Technology (ICT) underpins the DE. Broad application of ICT is referred to as informatization, which improves labor productivity [4]. DE development is helping to upgrade the industrial structure by improving production, cooperation, and innovation efficiencies [5]. The DE also establishes the digital retail market [6], with the leading role in driving China's current economic development.

Existing studies indicate that emissions trading systems can significantly reduce energy consumption by improving marketization, enhancing government-market relations and promoting the factor market development [7]. Urbanization increases energy consumption as rural populations relocate to cities and towns [8]. Rapid industrialization also increases energy consumption [8], and random shocks have transitory effects on energy consumption [9]. Furthermore, economic development and coal consumption are correlated [10], wherein increased local government economic intensity increases coal consumption [11].

In summary, no studies address DE's effect on coal consumption. Therefore, on the basis of the theoretical analysis and the provincial DE development index covering 30 provinces of China (excluding Xizang, Hong Kong, Macao, and Taiwan), this study uses the two-way fixed-effect (TWFE) model of individual and time to investigate the influence of DE development on coal consumption and the mechanism.

The possible contributions of this study are as follows. First, this study presents the first examination of the influence and mechanisms of DE development on coal consumption. Second, applying the mechanism analysis, this study examines the influence mechanism of DE development on coal consumption by using the mediating effect analysis, presenting a novel approach. Third, China has a long way to go to achieve the goals of carbon peaking and carbon neutrality. The conclusions of this study provide a new pathway for accomplishing that goal and new options for low-carbon development in many developing countries.

## 2 Theoretical analysis and research hypotheses

**Fig. 1:**
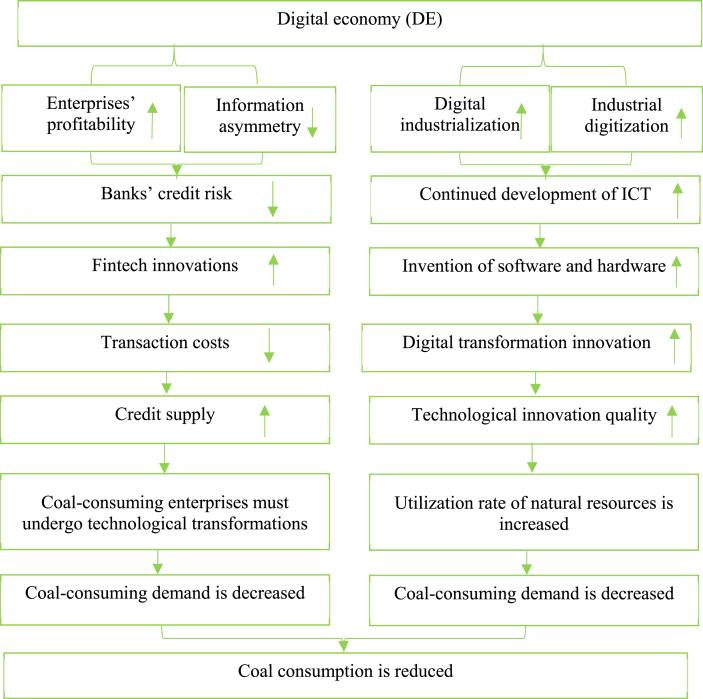
Mechanisms by which the DE affects coal consumption.

On the basis of the extant literature, we argue that the DE reduces coal consumption by influencing the fintech innovation level and quality of technological innovation through the mechanism shown in Fig. 1.

### 2.1 Mechanism analysis based on fintech

Financial technology (fintech) developed rapidly after the 2008 financial crisis. Countries across the globe strive to develop fintech, which has been reconstructing the global financial system. The development of fintech is inseparable from financial institutions as the primary bodies of innovation. Banks, which dominate the financial system in China, have a decisive role in fintech development, which subsequently depends on the banks’ willingness and ability to implement innovation.

DE development can effectively reduce banks' credit risk and improve the willingness and ability to implement fintech innovation. The mechanism lies is the DE's ability to improve the solvency of borrowing enterprises. First, DE development promotes the multidimensional cooperation of traditional production factors like labor and revolutionized social productivity [12]. Enterprises introduce a digital network into the production process to increase coordination efficiency [12], and total factor productivity (TFP) can be raised through the development of DE. Second, the DE development process refers to continuous implementation of digital transformation as enterprises increase the degree of informatization, which can improve labor productivity [4]. Subsequently improved TFP, labor productivity, and production efficiency enhance enterprises' profitability and borrowing solvency, advance bank credit asset quality, and reduce bank credit risk.

DE development also minimizes information asymmetry, which further reduces credit risk. Banks find it hard to judge potential borrowing enterprises’ real credit status and solvency due to severe information asymmetry; thus, credit risk is difficult for banks to effectively control prior to lending. After lending, effective and timely post-loan management are the key to preventing credit risk. Similarly, information asymmetry often makes it difficult for banks to accurately grasp the real use of capital and the follow-up debt circumstances of the borrowing enterprise or supervise enterprises to maintain solvency. DE, backed by big data and AI, can greatly decrease information asymmetries, reducing credit risk before and after lending.

In summary, DE development can reduce banks' credit risk, which improves banks' capabilities and willingness to carry out fintech innovations and foster further development of fintech in China. Introducing fintech into finance can reduce transaction costs (e.g., information acquisition costs) [13], which encourages banks to increase credit supply and eases coal enterprises' financing constraints. To comply with energy conservation and emissions reduction requirements, coal-consuming enterprises must undergo technological transformations after financing constraints are eased to reduce coal demand for the sake of long-term interests. Therefore, the development of fintech can alleviate coal-consuming enterprises' financing constraints and reduce coal demand and use. Therefore, this study argues that fintech development can decrease coal-consuming enterprises’ demand.

### 2.2 Mechanism analysis through technological innovation

Effective and quality technological innovation is essential for the development and survival of enterprises’ business activities. Yu et al. (2019) [14] measure technological innovation quality based on the proportion of discovery patents in all patent applications.

The DE can increase the quality of technological innovation via two mechanisms: digital industrialization and industrial digitization. Among them, digital industrialization is primarily embodied in invention and refers to the continued development of ICT [15]. Telecommunications industry development leads to the discovery of communication-related software (such as management software and operating systems) and hardware (such as chips), which embody typical substantive technological progresses (discovery). Creativity and intelligence are the fundamental characteristics of the current software industry [16]; therefore, ongoing software industry development and growth is accompanied by substantial ICT technological progress. Industrial digitization refers to the process by which enterprises employ big data, blockchain, AI, and other related technologies to conduct comprehensive transformation [17], which produces additional inventions.

Improved technological innovation quality significantly impacts coal-consuming enterprises’ coal demand. Greater technological progress by coal-consuming enterprises increases the utilization rate of natural resources, lowering coal demand. Therefore, this study argues that improved technological innovation quality lowers coal demand.

### 2.3 Hypotheses

In summary, DE development plays a role in stimulating fintech development and improving technological innovation quality, and the latter two reduce coal-consuming enterprises’ coal consumption. The following hypotheses are therefore proposed.

H1 DE development reduces coal consumption.

H2a DE development decreases coal consumption by facilitating fintech development.

H2b DE development reduces coal consumption through improving technological innovation quality.

## 3 Models and variables

### 3.1 Sample selection and data sources

After 2011, China's DE developed rapidly. For this reason, we use China's provincial-level data (excluding Xizang, Taiwan, Hong Kong, and Macao due to the missing data) over the period 2011–2022 to conduct our empirical testing. In 2022, Zero-One Thinktank and the Electronic Five Institute of the Ministry of Industry and Information Technology of the People's Republic of China jointly released a report on China's DE development index, providing data with good authority. As such, we obtain the provincial DE development index from Zero-One Thinktank (https://www.01caijing.com/) as a proxy variable for provinces' DE development.

We obtain the data of land area from Baidu, which is the largest search engine in China (https://zhidao.baidu.com/question/930384489837332579.html). National loan data are from the People's Bank of China (http://www.pbc.gov.cn/diaochatongjisi/116219/116319/4184109/4184113/index.html), which is China's central bank, and other data are from the Wind database (https://www.wind.com.cn/portal/zh/EDB/index.html) and the National Bureau of Statistics (NBS) (https://data.stats.gov.cn/easyquery.htm?cn=E0103). We processed the data as follows: (1) we linearly interpolate coal consumption for 2019–2022 because of missing data. (2) Linear interpolation is performed on loan balances and deposit balances in a few provinces for 2019–2022. (3) The study applies the two-tailed 1 % winsorization to all continuous variables to avoid the effects of outliers.

One of mediating variables variable in this study is fintech; however, since no data are publicly available regarding fintech development in China's provinces, we employ an alternative approach, referencing previous studies [[18], [19], [20]] by employing text mining techniques to generate the fintech development index encompassing various provinces as an estimate of fintech development.

Our methodology involves identifying relevant keywords and crawling unstructured text data from the ChinaDaily website (chinadaily.com.cn). The step-by-step process of fintech development index construction includes the following four steps:

The first step is constructing the keyword database. Fintech's core components are technology and innovation. While the innovations within the fintech sector can be categorized into innovations introduced by traditional financial institutions and those introduced by mew institutions. Consequently, we considered technology, fintech innovation within traditional financial institutions and those within new institutions when constructing the keyword database.

In the aspect of technology, we included the following keywords: “blockchain”, “AI”, “cloud computing”, and other keywords related to the fintech technology. Regarding fintech innovation within traditional institutions, keywords like “mobile banking” and “online banking” were incorporated. In the aspect of fintech innovation within new institutions, due to the reason that new institutions related to fintech mainly focus on payments and financing, the keywords like “mobile payment”, “third-party payment”, “online financing” and “online loan” were included. Notably, new institutions are often the first to implement emerging innovations, which are subsequently adopted by traditional institutions. Therefore, for the innovation keywords that both new and traditional institutions possess, we consider them as innovations in new institutions, which includes “internet banking”, “internet insurance”, “intelligent risk control”, and “internet securities”, and etc.

The second step is to obtain raw data. On the basis of multiple keywords of the three major types mentioned above, the process of generating raw data involved two primary steps. First, data collection in which initially unstructured text data were sourced from ChinaDaily's website were systematically crawled. Subsequently, an analysis of each keyword's frequency was conducted in every year during the sample period within each sample province in China using the province name + keyword format. Second, according to the three types of keywords phrases mentioned above, keyword frequencies were aggregated within each type.

The third step is getting the index weights. Based on the data acquired in the second step, we need to give each type of index an appropriate weight to get a single overall index. After the data standardization, the index weight was determined by the CRITIC method:

$$w_{i}=\frac{C_{i}}{\sum_{j}^{n}C_{j}},i=1,2\ldots ，n$$

where $C_{i}=\sigma_{i}\sum_{j}^{n}\left(1-r_{ij}\right),i=1,2\ldots n,$ which represent the i-th index, and *i* and *j* cannot take the same value at the same time, *r*_*ij*_ represent the correlation coefficient of the i-th and j-th indices, and *σ*_*i*_ represent the standard deviation of i-th index.

The fourth step is to yield the fintech development Index, which is an overall index of each province in every year during the sample period. We applied the weights calculated in the third step to the three types of index after the data standardization to generate the fintech development index (*fintechidx*). Considering that the minimum value of *fintechidx* is 0, we used the natural logarithm of (1 + *fintechidx*) to obtain the *fintech* and reduce the interference of heteroskedasticity.

### 3.2 Model design

#### 3.2.1 Model design for H1

Hypothesis H1 is tested using the TWFE model:

(1) $$coal_{it}=\alpha_{0}+\beta_{1}{dige}_{it}+\eta X_{1}+\theta_{i}+\delta_{t}+\varepsilon_{it}$$

Where i represents the province and t represents the year, $coal_{it}$ is the coal consumption, $\alpha_{0}$ is a constant term, $\theta_{i}$ represents the individual effect, $\delta_{t}$ represents the year effect, and $\varepsilon_{it}$ is the random disturbances. And ${dige}_{it}$ is the independent variable, which represents the digital economic development level, $\beta_{1}$ is its coefficient. For a significantly negative $\beta_{1}$, DE reduces coal consumption. $X_{1}$ denotes a list of control variables.

#### 3.2.2 Model design for H2

Hypothesis H2 is tested using the following model referencing [19]:

(2) $$coal_{it}=\alpha_{0}+\beta_{1}{dige}_{it}+\eta X_{1}+\theta_{i}+\delta_{t}+\varepsilon_{it}$$

(3) $$m_{it}=\alpha_{0}+\rho_{1}{dige}_{it}+\gamma X_{2}+\theta_{i}+\delta_{t}+\varepsilon_{it}$$

(4) $$coal_{it}=\alpha_{0}+\omega_{1}{dige}_{it}+\theta_{1}m_{it}+\eta X_{1}+\theta_{i}+\delta_{t}+\varepsilon_{it}$$

where $m_{it}$ is the mediating variable of fintech (fintech) and technological innovation quality (qinno) referencing Chen (2022b) [18]. $X_{1}$ in models (2) and (4) are same as in model (1), $X_{2}$ in model (3) is control variables (See below for details).

#### 3.2.3 Variable description

Table 1 describes the variable definitions that needed to test our hypotheses.

**Table 1:** Variable definitions.

	Variables	Symbols	Variable definitions
Dependent variable	Coal consumption	*coal*	Ln (coal consumption/number of legal persons in secondary industry)
		*rcoal*	Ln (coal consumption/number of legal persons in all industries) (robustness test)
Independent variable	Digital economy development	*dige*	The provincial DE development index obtained from the Zero-One Thinktank
		*rdige*	Ln(1+ *dige*)
Control variable	Economic development	*pgdp*	Log(Real GDP per person)
	Industrial structure development	*struct*	1 * primary industry's proportion + 2 * secondary industry's proportion + 3 * tertiary industry's proportion
	R&D investment	*RD*	R&D investment of industrial enterprises above large scale/GDP
	Government intervention	*govint*	Fiscal expenditure/GDP
	Financial decentralization	*fide*	Loan balance in each province/national loan balance * 100
	Financial efficiency	*feff*	Loan balance/deposit balance collected from the WIND Database
	Population density	*popd*	Each province's population/each province's land area
	Environmental pollution	*poll*	Industrial sulfur emissions/total population
	Financial development	*fide*	Each province's loan balance/its GDP
	Economic openness	*pfdi*	Ln(real FDI)
	Population size	*pop*	Ln (total population)
	Financial investment in technology	*rexpen*	Local fiscal expenditure on technology/GDP

##### 3.2.3.1 Dependent variable

The dependent variable is coal consumption (*coal*), of which the secondary industry accounts for 98.4 % [10]. Taking natural logarithms can reduce heteroskedastic interference. Here, coal consumption is divided by the number of legal entities in the secondary industry, the natural logarithm is used to represent coal consumption (*coal*), and the natural logarithm of coal consumption divided by the number of legal entities in the all industries (*rcoal*) is used for robustness test.

##### 3.2.3.2 Independent variables

**Fig. 2:**
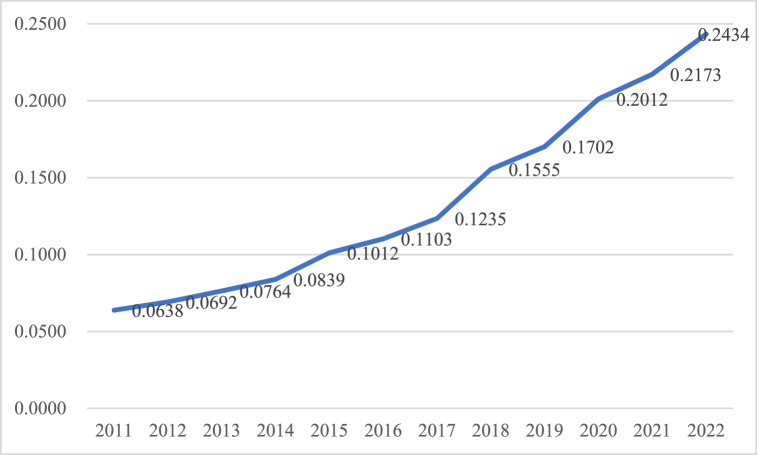
The average of China's provincial-level DE development index.

The independent variable was the DE development index (*dige*), which is obtained from the Zero-One Thinktank as a proxy variable. And *rdige* was used for robustness check, whose measurement is shown in Table 1. Fig. 2 shows the average of China's provincial DE development index (*dige*) from 2011 to 2022. From Fig. 2, China's DE has a continuously growing trend, with a faster growth rate after 2017.

##### 3.2.3.3 Mediating variables

1. Fintech level (*fintech*)

Referencing Chen et al.(2022b) [18], Chen(2023) [19], and Li and Cheng(2023) [20], this study uses the text mining approach described above to construct a fintech development index (*fintechidx*). Considering that the minimum value of *fintechidx* is 0, we use the natural logarithm of (1 + *fintechidx*) to obtain the fintech index measure (*fintech*) and reduce the interference of heteroskedasticity.

2. Quality of technological innovation (*qinno*)

Invention patents are the most innovative among all patents, which include invention patents, utility models, and designs; therefore, we quantify the amount of invention patents per capita, and take it as a proxy variable for technological innovation quality.

##### 3.2.3.4 Control variables

Referencing previous studies [5,10,11,21], the control variables of equation (1) include economic development (*pgdp*), industrial structure development (*struct)*, R&D investment (*RD*), government intervention (*govint*), financial decentralization (*fide*), financial efficiency (*feff*), population density(*popd*), and environmental pollution (*poll*).

The control variables of equation (3) are as follows:

When the fintech level (*fintech*) is the dependent variable, the control variables include economic development (*pgdp*), financial development (*fide*), economic openness (*pfdi*), financial efficiency (*feff*), financial decentralization (*fide*), and government intervention (*govint*).

When technological innovation (*qinno*) is the dependent variable, the control variables include economic development (*pgdp*), economic openness (*pfdi*), financial development (*fide*), financial efficiency (*feff*), fiscal technology expenditure (*rexpen*), and population size (*pop*).

## 4 Empirical analysis

### 4.1 Descriptive statistics

The descriptive statistics of the variables are presented in Table 2, revealing that the average, minimum, and maximum values of digital economic development (*dige*) were 0.1347, 0.0792, and 0.4488, respectively. This is because development in China is unbalanced, with a large gap in DE development among provinces. Furthermore, digital economic development (*dige*) and other variables includes data from 30 provinces from 2011 to 2022, with 360 observations.

**Table 2:** Descriptive statistics of main variables.

Variable	Observations	Mean	Standard deviation	Minimum	Maximum
*coal*	360	4.8103	1.0913	0.7084	7.2176
*rcoal*	360	5.6683	1.2042	0.2944	8.1730
*dige*	360	0.1347	0.0792	0.0345	0.4488
*rdige*	360	0.1240	0.0667	0.0339	0.3707
*fintech*	360	0.0713	0.0912	0.0011	0.4789
*qinno*	360	0.7587	1.0566	0.0513	8.6629
*pgdp*	360	1.6086	0.4281	0.4643	2.7276
*struct*	360	3.6534	0.8560	2.6691	8.1162
*RD*	360	1.1125	0.6032	0.1663	3.2416
*govint*	360	0.2584	0.1085	0.1050	0.7534
*fide*	360	0.0308	0.0231	0.0023	0.1061
*feff*	360	0.7971	0.1567	0.4085	1.2278
*popd*	360	0.4756	0.7104	0.0080	3.9508
*poll*	360	1.0005	1.1332	0.0087	6.3333

### 4.2 Benchmark regression

For equation (1), both fixed effects (FE) and random effects (RE) were examined for potential estimation and the Hausman test was performed in this study. The chi-square statistic of Hausman test was 69.53, with the p-value less than 0.0001, indicating FE should be used. When conducting the estimation, we gradually added control variables to equation (1) and Table 3 presents the results.

**Table 3:** Fixed-effect estimation results of equation (1).

	(1)	(2)	(3)	(4)	(5)
Variable	*coal*	*coal*	*coal*	*coal*	*coal*
*dige*	− 2.441***	−2.423***	−2.497***	−2.707***	−2.670***
	(0.707)	(0.681)	(0.674)	(0.634)	(0.744)
*pgdp*		−0.983**	−0.728	−0.541	−0.0692
		(0.432)	(0.467)	(0.476)	(0.496)
*struct*		−0.0805	−0.179	−0.188	−0.141
		(0.135)	(0.156)	(0.153)	(0.161)
*RD*		−0.0664	−0.118*	−0.0984	−0.152*
		(0.0668)	(0.0700)	(0.0691)	(0.0844)
*govint*			1.738**	1.716**	2.119***
			(0.739)	(0.711)	(0.683)
*fide*				−7.204**	−6.512**
				(2.888)	(2.737)
*feff*				0.0565	0.107
				(0.277)	(0.269)
*popd*					1.767
					(1.680)
*poll*					−0.0768***
					(0.0240)
*Constant*	5.430***	6.955***	6.585***	6.579***	5.087***
	(0.0754)	(0.611)	(0.603)	(0.573)	(1.082)
Year FE	YES	YES	YES	YES	YES
Province FE	YES	YES	YES	YES	YES
Observations	360	360	360	360	360
N	30	30	30	30	30
R^2^	0.731	0.741	0.747	0.751	0.758

Note: * * *, * *, and * denote significance levels of 1 %, 5 %, and 10 %, respectively; double clustered time and individual robust standard errors are in brackets. The same below.

#### 4.2.1 Research hypothesis testing

Columns (1)–(5) of Table 3 reveal that the *dige's* coefficients are significantly negative at 1 % significance levels, confirming that DE development reduces coal consumption, which validates hypothesis H1.The descriptive statistics in Table 2 and column (5) of Table 3 reveal that for each standard deviation increase in DE, coal consumption decreases by 0.1938.

**Fig. 3:**
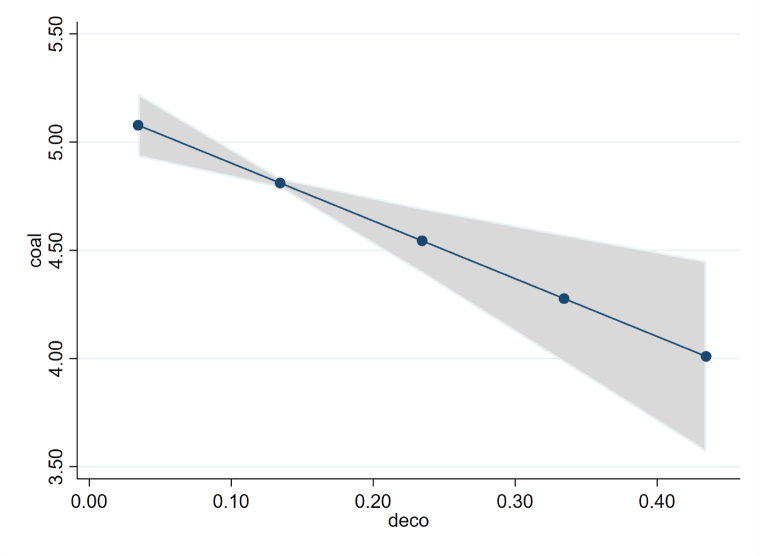
Marginal impact of the DE on coal consumption.

Fig. 3 shows the marginal impact of the DE on coal consumption on the basis of column (5) of Table 3. As seen in Fig. 3, coal consumption continues to decline as the level of DE development increases.

#### 4.2.2 Other information

As we can see from Column (5) of Table 3, first, the coefficient of government intervention (*govint*) is significantly positive, which may be attributable to increased economic intensity of local government intervention that raises coal consumption [11]. Second, the coefficient of *fide* is significantly negative, perhaps because local governments have used financial decentralization to provide credit funds to coal-using enterprises for technological transformation to reduce environmental pollution from coal consumption. Third, the coefficient of environmental pollution (*poll*) is significantly negative at 1 % significance level. The rationale for this may be local governments implementing measures to reduce coal consumption to control environmental pollution and fulfill the central government's promotion of energy conservation and emissions reduction. The coefficients of the remaining control variables are basically not significant.

### 4.3 Robustness tests

We have applied the double clustering standard error in columns (1)–(5) of Table 3 in order to increase the reliability of the estimates, as it can overcome the influence of autocorrelation and heteroskedasticity [5], revealing that the independent variables are significant. So, this is a robustness test and confirms the robust of H1's conclusion. We also used the following additional method to conduct robustness tests.

#### 4.3.1 Endogenous problems

DE development affects coal consumption; however, coal combustion also produces sulfur dioxide, which pollutes the environment; therefore, coal-consuming enterprises face pressures for energy conservation and emissions reduction. Digital transformation is a choice for enterprises to respond to governmental pressure, which also promotes DE development. Therefore, coal consumption may also affect DE development (*dige*), representing a bidirectional causal relationship between DE development and coal consumption, making *dige* variable endogenous.

Referencing Kim et al. (2014) [22], we get an instrumental variable *ivdige* by caculating the mean of other provinces’ DE development in the same year. Then equation (1) is estimated with *ivdige* as the instrumental variable (IV) using the IV method, resulting in in column (1) of Table 4. The estimation yield a Cragg–Donald F statistic of 3162.392, and the statistic is used to check if the IV is weak IV. The value of 3162.392 far exceeds the critical value of 16.38 under a 10 % error, confirming that *ivdige* is an effective IV. Therefore, the IV results confirming that DE development reduces coal consumption when excluding endogeneity. So, we conclude that H1 remains robust.

**Table 4:** Robustness test results of equation (1).

	(1)	(2)	(3)	(4)	(5)
Variable	*coal*	*rcoal*	*coal*	*coal*	*coal*
*dige*	−2.559***	−3.122***		−2.670***	−2.670***
	(0.702)	(0.790)		(0.744)	(0.551)
*rdige*			−3.589***		
			(0.988)		
*t*				−0.0616*	
				(0.0367)	
Year FE	YES	YES	YES	YES	YES
Province FE	YES	YES	YES	YES	YES
Control variables	YES	YES	YES	YES	YES
Observations	360	360	360	360	360
N	30	30	30	30	30
R^2^	0.963	0.845	0.758	0.758	–

#### 4.3.2 Replacing the dependent variable

We get *rcoal* using the method mentioned in Table 1, and taking *rcoal* as the dependent variable to do the robustness test. Equation (1) is re-estimated using FE, resulting in column (2) of Table 4. The results confirm that DE development reduces coal consumption.

#### 4.3.3 Replacing the independent variable

We get *rdige* using the method mentioned in Table 1, and taking it as the independent variable. Then we conducted estimation of equation (1) using FE, presenting the results in column (3) of Table 4. The results confirm that DE development reduces coal consumption.

#### 4.3.4 Controlling the continuous time variable

The Chinese government has been promoting energy conservation and emissions reduction for several years, which means that coal consumption may exhibit a time trend. Therefore, we added continuous time variable (*t*) when conducting the estimation. The results are presented in column (4) of Table 4, which also confirm that DE development reduces coal consumption.

#### 4.3.5 Changing the estimation method

The maximum likelihood estimation method is used to re-estimate equation (1), as this method can overcome the bias introduced by the estimation method, and the results are presented in column (5) of Table 4.

Overall, we conclude from all the results in Table 4 that the conclusion of H1 is robust.

## 5 Mechanism tests

In section 2, we analyzed that the DE theoretically reduces coal consumption by influencing the development of fintech and technological innovation quality. We used the mediating effect analysis to test the mechanism to verify research hypotheses H2a and H2b.

### 5.1 Mechanism test examining fintech

With fintech development (*fintech*) as the mediating variable, we used FE to estimate equations (2), (3), (4), presenting the results in paths A–C in Table 5, Panel A.

**Table 5:** Mechanism test results.

	Panel A		
	path A	path B	path C
Variable	*coal*	*fintech*	*coal*
*dige*	−2.670***	1.117***	−1.902***
	(0.744)	(0.120)	(0.711)
*fintech*			−0.890*
			(0.539)
Year FE	YES	YES	YES
Province FE	YES	YES	YES
Control variables	YES	YES	YES
Observations	360	360	360
N	30	30	30
R^2^	0.758	0.754	0.762
	*Panel B*		
	path A	path B	path C
*Variable*	*coal*	*qinno*	*coal*
*dige*	−2.670***	5.999***	−1.450**
	(0.744)	(1.372)	(0.581)
*qinno*			−0.318***
			(0.0532)
Year FE	YES	YES	YES
Province FE	YES	YES	YES
Control variables	YES	YES	YES
Observations	360	360	360
N	30	30	30
R^2^	0.758	0.514	0.807

First, the coefficient of *dige* is significant at the 1 % level (seen in path A in Table 5, Panel A). So, we can conclude that there is a total effect. Second, the coefficient of digital economy development (*dige*) is significant positive (seen in path B in Table 5, Panel A), indicating that DE development promotes fintech development. Third, the coefficient of the fintech development is significant negative, the coefficient of digital economy development (*dige*) is significant negative (seen in path C in Table 5, Panel A). Therefore, we can conclude that *fintech* plays a part mediating role. The DE progress facilitates fintech development, which reduces coal consumption, confirming that hypothesis H2a is valid.

### 5.2 Mechanism test based on technological innovation

Equations (2), (3), (4) are estimated with coal consumption (*coal*) as the dependent variable and technology innovation quality (*qinno*) as the mediating variable, presenting the results under path A, B, and C in Table 5, Panel B.

Similarly, Panel B of Table 5 confirms that hypothesis H2b was valid.

In summary, DE development affects coal consumption by promoting fintech development and improving technological innovation quality.

## 6 Discussion

This study confirmed that DE development can reduce coal consumption. Digital technologies such as AI are key technologies for advancing DE development [18,23]. AI automates tasks that were previously performed by the workforce [24], which saves enterprises’ production and operation time, improves decision making, optimizes processes, and enhances enterprise efficiency [25,26]. This means that resulting from DE development, coal-consuming enterprises can use AI technology to improve efficiency and reduce coal consumption. AI can also be used to quickly and accurately predict and analyze processes to help enterprises develop energy plans, improve energy efficiency, and reduce coal consumption [27]. Concerning the key technologies for DE development, our findings are consistent with the conclusions deduced from these studies. In addition, electricity and coal are major sources of energy and emissions. Cheng and Chen (2023) [28] find that DE development has the effect of reducing electricity consumption intensity, and our findings are consistent with those of Cheng and Chen (2023) [28].

Based on digital technologies such as AI, fintech innovations advance the online presence and intelligentization of financial services [29], expanding the boundaries of financial services [30], which consequently intensifies competition among banks and raises banks’ informed risk-taking [18], prompting the provision of more loans. Enterprises can use such funds to upgrade and purchase high-tech environmental protection equipment. As a result, fintech can improve energy efficiency [31,32], spurring economies to transition from traditional energy sources to renewable sources such as hydro, wind, geothermal, and solar power [33]. Our findings are in line with previous studies, and we also confirmed that DE development can facilitate fintech development.

## 7 Conclusion and prospects

This study used the TWFE model to examine the influence and mechanism of DE development on coal consumption. The results demonstrated that DE development reduces coal consumption. We also found that DE development reduces coal consumption by driving fintech development and improving technological innovation quality.

This study has some policy implications. **First**, DE development is correlated with reduced coal consumption, which is one of the major sources of carbon dioxide emissions. Therefore, to promote economic development and reduce carbon emissions, it is essential for developing countries to actively promote DE development. **Second**, fintech development is also correlated with reduced coal consumption. Developing and developed countries alike can reduce coal consumption and carbon dioxide emissions by promoting fintech development. Third, DE development can improve technological innovation quality. Sci-tech innovation is of great significance to developed and developing countries; therefore, all countries should promote DE development to improve technological innovation quality and contribute to economic high-quality development.

Due to the lack of relevant statistical mechanisms, we conducts an empirical analysis using provincial DE development index, rather than a more detailed study using a local municipal DE development index. So this is a limitation of this study. We focused on only two mediators of fintech and technological innovation quality, and other paths in the DE could affect coal consumption. It was difficult to exhaust all paths in this study, which is another limitation.

The conclusions of this study are based on provincial data from China. Whether this conclusion can be directly applied to prefecture-level cities remains to be investigated. The reason is that China is not only a vast country, but also characterized by uneven regional development, with differences in DE development among prefecture-level cities. This phenomenon exists among different cities in the same province. For example, the DE of Guangdong Province is an extremely developed, and Guangzhou and Shenzhen in this province also have high DE development levels, whereas other prefectural-level cities have relatively low DE development levels. For cities with a low DE development levels, whether DE development has the effect of supporting fintech development or improving enterprises’ technological innovation quality requires further investigation. In addition, another future line of research could continue to explore other path through which DE development affects coal consumption.

## Ethical Statements

Ethic Approval.

Our institution does not require ethics approval for reporting individual cases or case series.

## Data availability statement

Data will be made available on request.

## CRediT authorship contribution statement

**Qijing Xiong:** Writing – review & editing, Writing – original draft, Supervision, Software, Methodology, Formal analysis, Conceptualization. **Peter Chan:** Writing – review & editing, Writing – original draft, Software, Conceptualization. **Fan Bai:** Writing – review & editing, Writing – original draft.

## Declaration of competing interest

The authors declare that they have no known competing financial interests or personal relationships that could have appeared to influence the work reported in this paper.
